# Genetic connectivity across marginal habitats: the elephants of the Namib Desert

**DOI:** 10.1002/ece3.2352

**Published:** 2016-08-03

**Authors:** Yasuko Ishida, Peter J. Van Coeverden de Groot, Keith E. A. Leggett, Andrea S. Putnam, Virginia E. Fox, Jesse Lai, Peter T. Boag, Nicholas J. Georgiadis, Alfred L. Roca

**Affiliations:** ^1^ Department of Animal SciencesUniversity of Illinois at Urbana‐ChampaignUrbanaIllinois61801; ^2^ Department of BiologyQueen's UniversityKingstonONK7L 3N6Canada; ^3^ Namibian Elephant and Giraffe TrustOutjoNamibia; ^4^ Department of Life SciencesSan Diego Zoo GlobalSan DiegoCalifornia92112; ^5^ Elephant Human Relations Aid (EHRA)SwakopmundNamibia; ^6^ Puget Sound InstituteUniversity of WashingtonTacomaWashington98421; ^7^ Carl R. Woese Institute for Genomic BiologyUniversity of Illinois at Urbana‐ChampaignUrbanaIllinois61801; ^8^ Present address: Fowlers Gap Arid Zone Research StationSchool of Biological, Earth and Environmental SciencesUniversity of New South WalesKensingtonNSW2051Australia

**Keywords:** Etosha National Park, *Loxodonta africana zukowski*, microsatellites, mitochondrial DNA

## Abstract

Locally isolated populations in marginal habitats may be genetically distinctive and of heightened conservation concern. Elephants inhabiting the Namib Desert have been reported to show distinctive behavioral and phenotypic adaptations in that severely arid environment. The genetic distinctiveness of Namibian desert elephants relative to other African savanna elephant (*Loxodonta africana*) populations has not been established. To investigate the genetic structure of elephants in Namibia, we determined the mitochondrial (mt) DNA control region sequences and genotyped 17 microsatellite loci in desert elephants (*n *=* *8) from the Hoanib River catchment and the Hoarusib River catchment. We compared these to the genotypes of elephants (*n *=* *77) from other localities in Namibia. The mtDNA haplotype sequences and frequencies among desert elephants were similar to those of elephants in Etosha National Park, the Huab River catchment, the Ugab River catchment, and central Kunene, although the geographically distant Caprivi Strip had different mtDNA haplotypes. Likewise, analysis of the microsatellite genotypes of desert‐dwelling elephants revealed that they were not genetically distinctive from Etosha elephants, and there was no evidence for isolation by distance across the Etosha region. These results, and a review of the historical record, suggest that a high learning capacity and long‐distance migrations allowed Namibian elephants to regularly shift their ranges to survive in the face of high variability in climate and in hunting pressure.

## Introduction

Substantial phenotypic and genotypic variation can often be found in species with large geographic ranges, with intraspecies differences often following spatial and environmental gradients (Lesica and Allendorf 1995). For species with continuous distributions covering an environmental gradient, higher phenotypic plasticity is expected to evolve at the edges of the geographic range, which can enable the invasion of new habitats (Chevin and Lande 2011). Viability and reproductive success are often reduced in marginal environments (Kawecki 2008), affected by lower abundance of the species and by reduced environmental suitability in the periphery (VanDerWal et al. 2009; Martínez‐Meyer et al. 2013; Edwards and Derocher 2015).

Peripheral populations may face selective pressure different from core populations due to severe environmental conditions that can affect the quality and quantity of food, water, and other resources. The ability to eventually adapt to marginal habitats, in which survival and reproduction may initially be poor, plays a crucial role in the expansion of ecological niches and of geographic range for a species (Kawecki 2008; Sutter and Kawecki 2009). Some conditions may favor local isolation and adaptation to marginal habitats, making marginal populations less demographically and genetically dependent on core habitats and less prone to the gene flow from core populations that counteracts the effects of local selection (Kawecki 2008). Populations living in marginal habitats at the peripheries of the species distribution would be of increased conservation concern if they have become genetically distinctive (Lesica and Allendorf 1995; Hampe and Petit 2005). Due to isolation and low numbers, peripheral populations may be more imperiled than core populations (Lesica and Allendorf 1995; Kawecki 2008).

The elephants of the Namib Desert represent an opportunity to examine the evolution of a peripheral population of the African savanna elephant (*Loxodonta africana*). The existence of elephants in the Namib Desert was first documented as early as 1793 (Viljoen 1992; Gröning 1998). They comprise one of the few populations of African savanna elephants that live in desert habitats (Viljoen 1989b). Elephants are unspecialized feeders and are highly mobile (Viljoen 1992). Elephants also have a keen understanding and memory of the spatial properties of their ecosystems relative to their location at any given time (Polansky et al. 2015). The ability of species such as elephants to learn and change their behavior can limit the need to genetically adapt to a new environment and can allow species to expand their range to novel marginal habitats that differ sharply from core habitats (Sutter and Kawecki 2009). Learning may allow a population to favor a new habitat specialization in a novel environment, thus increasing its isolation from other populations (Beltman and Metz 2005). Paradoxically, learning can also buffer against the effects of natural selection, thereby lowering the pressure for local genetic adaptations to develop (Sutter and Kawecki 2009).

Behavioral adaptations to their environment have been documented among desert elephants. They were reported to travel for up to 4 days without drinking water to access food sources as far as 70 km from waterholes (Viljoen 1989b, 1992) and also routinely engage in thermoregulatory behaviors that are rarely used by elephants elsewhere (Leggett 2004). Desert elephant survival is believed to depend on their intimate knowledge of the distribution of resources within their home ranges, which are considerably larger than those of elephants elsewhere (Viljoen 1989b). While an initial study of desert elephants found no evidence of migration into or out of their habitat in the northern Namib Desert (Viljoen 1989b), a subsequent study reported seasonal movement by a bull elephant between desert regions and Etosha National Park (NP), suggesting that gene flow may be possible between desert elephants and those of other regions (Leggett 2006, 2010). This would suggest that, despite the earlier report that migration did not connect desert elephants to other populations (Viljoen 1989b), desert elephants may not be genetically isolated from other savanna elephant populations.

On the other hand, a number of phenotypic differences have been attributed to desert elephants. They are said to be taller with a leaner build, longer legs and larger feet than other savanna elephants (Gröning 1998). Based on a purportedly more circular ear shape than other savanna elephants, the elephants of the Kaokoveld have even been described as a separate subspecies, *L. africana zukowsky* (Strand 1924). Desert elephants would thus represent an opportunity to examine the role of local genetic adaptation, relative to the role of learning and behavioral changes, in the ability of a species to occupy an extreme environment. Should they prove to be genetically distinct, this would also raise their conservation importance (Lesica and Allendorf 1995). We therefore examined Namibia's desert elephants using molecular genetic markers. In particular, we wanted to determine the degree of genetic diversity present among desert‐adapted elephants and whether they formed a population that was genetically different from savanna elephants elsewhere in Namibia and the rest of Africa.

## Materials and Methods

### Sample collection and geographic groupings

This study was conducted in compliance with the University of Illinois Institutional Animal Care and Use Committed approved protocol number 12040. Samples were obtained in full compliance with Convention on International Trade in Endangered Species of Wild Fauna and Flora and other required permits. From desert elephants, four blood samples were collected from the Hoarusib River catchment, and 14 dung samples were collected in the Hoanib River catchment. From other Namibian elephants, tissue samples were collected from 60 elephants in or near Etosha NP and the Huab River catchment (skin in salt‐saturated DMSO, skin biopsies, dried skin or dried skull tissue); only one individual was sampled from any herd encountered, and the distinctiveness of each elephant was subsequently verified after genotyping. Dung samples were collected from an additional 24 elephants in or near Etosha, the Huab River catchment, and the Ugab River catchment. Multiple samples of dung were sometimes collected from the same individual, but each individual was included only once in analyses to avoid overcounting.

**Figure 1:**
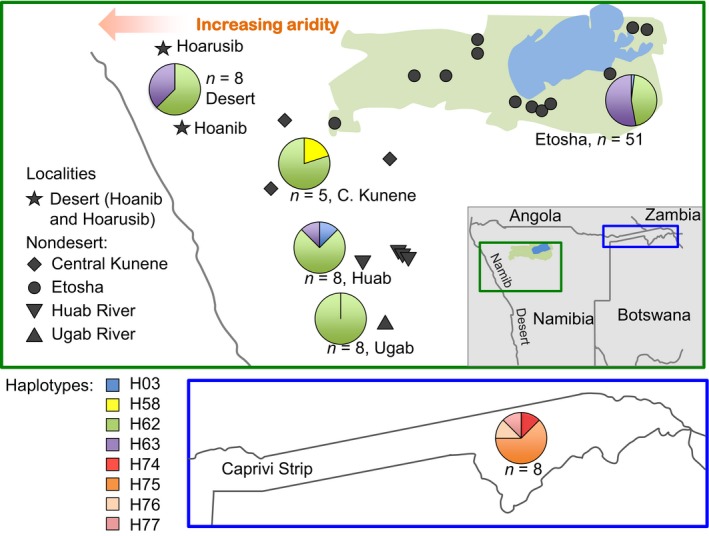
Map showing the sampling locations for Namibian elephants and the mtDNA haplotype distributions. The Namibian elephant samples were grouped geographically into six regions, each indicated by a different icon shape in black. The top panel has a shaded inset map showing the location of Namibia within southwestern Africa and the location of the Namib Desert (along the shoreline of the Atlantic Ocean). In the top panel, Etosha National Park is shown as light green shading, with Etosha Pan in blue. The two localities with desert‐dwelling elephants are the Hoarusib River catchment and the Hoanib River catchment. The lower panel shows the Caprivi Strip region of northeast Namibia that is surrounded by Angola, Botswana, Zambia, and Zimbabwe. Pie charts show the frequencies of 316 bp of mtDNA control for each locality. Note the similarity of desert elephants and those of other localities, but the distinctiveness of mtDNA haplotypes in the Caprivi Strip.

Elephants within Namibia were placed into up to six geographically defined groups. One of these was the Caprivi Strip, a narrow geographic protrusion of Namibia that is surrounded by Angola, Botswana, Zambia, and Zimbabwe (Fig. 1). Elephant sequences and haplotype frequencies for Caprivi were available from a previous study (Nyakaana et al. 2002). Our Namibian elephants were from outside Caprivi, and we placed them into two or into five geographically defined groups, as noted for each analysis below. The elephants from the Hoanib River catchment and from the Hoarusib River catchment were placed into a desert elephant group. Elephants from other Namibian localities (outside Caprivi) were placed into a single “nondesert” category for some of the analyses. For other analyses, our Namibian elephants that were not desert dwelling (and not from Caprivi) were placed into these geographically defined groups: Etosha (consisting of elephants within Etosha NP), Central Kunene (three locations southwest of Etosha NP), Ugab (elephants from the Ugab River catchment), and Huab (elephants from the Huab River catchment). Figure 1 shows the distribution of collection sites across northern Namibia, while information on our samples is listed in Table S1. Some analyses only involved elephants for which precise geographic coordinates were available.

### DNA extraction, PCR, and sequencing of mitochondrial DNA

The QIAamp DNA Stool Mini Kit (Qiagen, Valencia, CA) was used to extract DNA from samples of dung; while for other types of samples, phenol–chloroform extraction or the QIAamp DNA Mini Kit (Qiagen) was used. Steps were taken to minimize the possibility of amplifying numts, as described previously (Roca et al. 2007), with primers designed to target only regions of mtDNA conserved across elephant species. For high‐quality DNA, the primers and PCR methods used were as previously described for generating 4258 bp of continuous mtDNA sequence from part of the *MT‐ND5* gene through part of the control region (Ishida et al. 2013). PCR (and sequencing) of dung‐extracted DNA used primers CR‐F11 (5′‐TTACATGAATTGGCAGYCAACC) and CR‐R11 (5′‐AGATGTCTTATTTAAGAGGAAAG), with final concentrations of 0.4 μmol/L of each oligonucleotide primer, 1.5 mmol/L MgCl_2_, 200 μmol/L of each of the dNTPs (Applied Biosystems Inc. [ABI], Life Technologies Corp., Carlsbad, CA), 1× PCR Buffer II (ABI), and 0.04 units/μL final concentration of AmpliTaq Gold DNA polymerase (ABI). PCR consisted of an initial denaturation at 95°C for 9:45 min, with cycles of 20‐sec denaturation at 94°C, followed by 30‐sec annealing at 60°C (3 cycles); 58, 56, 54, or 52°C (5 cycles each temperature); or 50°C (last 22 cycles), followed by 75‐sec extension at 72°C, with a final extension of 3 min at 72°C. A negative (water) control was included with each PCR; no cross‐contamination was ever detected. PCR products were enzyme‐purified with Exonuclease I (USB Corporation, Santa Clara, CA) and shrimp alkaline phosphatase (USB Corporation). Sequences in both directions were generated using the BigDye Terminator system (ABI) as previously described (Ishida et al. 2013), purified with Sephadex G‐50 (Amersham, UK), and resolved on an ABI 3700 DNA Sequencer, or purified and resolved on an ABI 3730XL capillary sequencer. Sequencher (Gene Codes Corporation, Ann Arbor, MI, USA) was used to examine, edit, and concatenate sequences. Gene identity was established using BLAST (http://www.ncbi.nlm.nih.gov/blast/Blast.cgi; Zhang et al. 2000).

### Network and population genetic analyses of mitochondrial haplotypes

Median‐joining (MJ) networks were constructed using the software Network 4.6.1.1 (Bandelt et al. 1999) for both a 4258‐bp sequence dataset and a 316‐bp control region sequence dataset. The networks combined novel sequences with those of previous trans‐continental studies of African elephants, including previously generated sequences from the Etosha region and the Caprivi Strip in Namibia (Eggert et al. 2002; Nyakaana et al. 2002; Debruyne et al. 2003; Debruyne 2005; Johnson et al. 2007; Ishida et al. 2011, 2013), along with novel Namibian sequences. For the 4258‐bp dataset, haplotype sequences were known for all locations and were indicated on the network. However, some previous studies that used control region sequences did not report their frequencies across populations. Thus, the network generated using 316‐bp control region sequences did not show haplotype frequencies. Arlequin version 3.5.1.3 (Excoffier and Lischer 2010) was used to calculate *F*_ST_ between locations within Namibia, using 316‐bp control region sequences, with *P* values estimated using 10,000 permutations. Arlequin was also used to conduct exact tests of population differentiation using a Markov chain with 1,000,000 steps.

### Microsatellite genotyping

We initially attempted to genotype the elephants at 22 microsatellite loci; these markers are listed in Table S2 (Nyakaana and Arctander 1998; Comstock et al. 2000, 2002; Fernando et al. 2001; Archie et al. 2003; Ishida et al. 2011). For genotyping, only DNA samples extracted from tissue or blood were used. Previously published protocols were followed for the PCR setup and algorithm (Menotti‐Raymond et al. 2005; Ishida et al. 2011) and for fluorescent labeling (Boutin‐Ganache et al. 2001). The PCR algorithm consisted of an initial 95°C for 10 min, with cycles of 15 sec at 95°C, followed by 30 sec at 60, 58, 56, 54, 52°C (2 cycles at each temperature), or 50°C (last 30 cycles); and 45 sec at 72°C, with a final extension of 30 min at 72°C (Menotti‐Raymond et al. 2005; Ishida et al. 2011, 2012). Samples were genotyped on an ABI 3730XL capillary sequencer and analyzed with Genemapper version 3.7 (ABI).

### Analyses of microsatellite data

Microsatellite data were tested for significant deviation from Hardy–Weinberg equilibrium (HWE) and for linkage disequilibrium (LD) using Genepop 4.2 (Rousset 2008). A Markov chain algorithm was used to test for HWE using 10,000 dememorization steps, 200 batches and 1000 iterations per batch. LD was tested using 1000 dememorization steps, 100 batches and 1000 iterations per batch for each combination of loci. Of 22 loci genotyped, five were not used in subsequent analyses. Marker EMX‐5 was monomorphic among the Namibian elephants and was removed for this reason. Those loci found to deviate significantly (after Bonferroni correction) from HWE or LD also were not used for subsequent analyses. Microsatellites LaT06 and FH19 were not used as they showed significant deviation from HWE after Bonferroni correction (*P *<* *0.002). Loci LAF29 and FH39, and loci LAF37 and FH127, were found to be in significant LD after Bonferroni correction (210 tests, *P *<* *0.0002). In each pair, the locus with fewer alleles (LAF29 and LAF37, respectively) was excluded because of the LD.

Arlequin version 3.5.1.3 (Excoffier and Lischer 2010) was used to calculate expected heterozygosity (*H*_e_) and observed heterozygosity (*H*_o_) and to estimate *F*_ST_, using 10,000 permutations to test for significance. Arlequin was also used for exact tests of population differentiation, using 1,000,000 steps in the Markov chain, after 100,000 steps of dememorization. GenAlEx 6.5 (Peakall and Smouse 2012) was used to independently calculate *F*_ST_ and used for principal coordinates analyses (PCoA) and for tests of spatial autocorrelation. These analyses were performed to explore whether ecotypes represent natural genetic groupings. For the spatial autocorrelation analyses, nonstandardized pairwise interindividual genetic distances were calculated for each sample pair. Geographic distance for each pair was also calculated for samples for which precise geographic location was available. The spatial autocorrelation analysis divided the pairwise distances into five equal classes, and the statistical significance of the autocorrelation coefficient (*r*) was tested with 999 random permutations and 1000 bootstrap iterations. STRUCTURE 2.3.4 (Pritchard et al. 2000), which applies a model‐based clustering algorithm to multilocus genotype data, was used to infer population structure. STRUCTURE was run three times for each value of *K* from 1 to 10, without the use of prior information on locality, under the admixture‐correlated model, with each iteration using at least 1 million Markov chain Monte Carlo generations following a burn‐in of at least 100,000 steps.

## Results

### Mitochondrial DNA analyses

**Figure 2:**
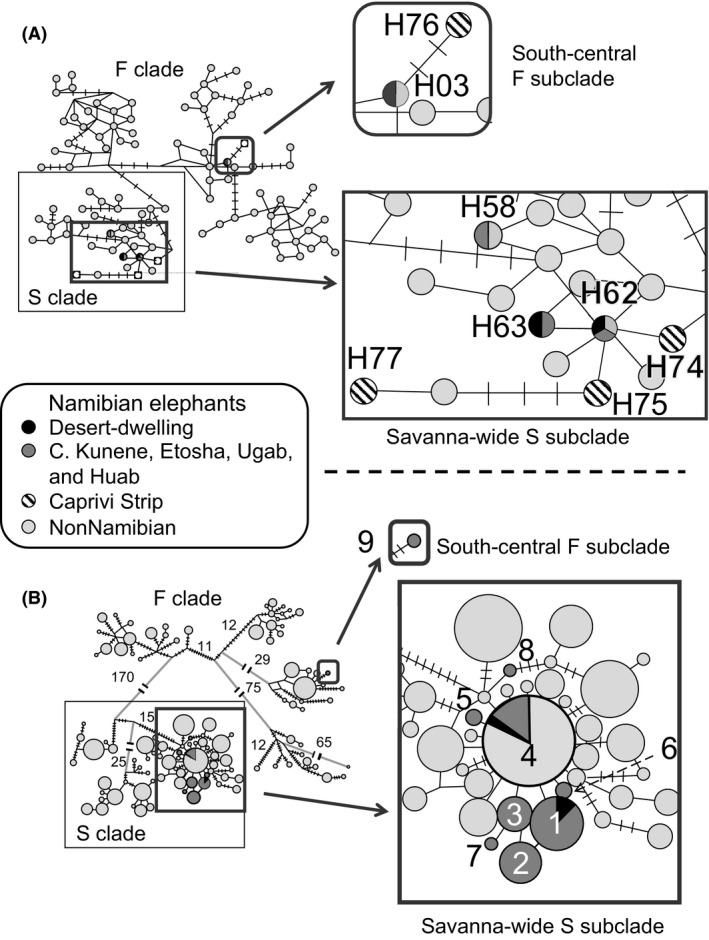
Networks showing the relationships among mitochondrial haplotype sequences of elephants from Namibia and across Africa. The median‐joining networks (Bandelt et al. 1999) were generated using alignments of newly generated and previously published sequences of (A) 316 bp of control region sequences and (B) 4258 bp mtDNA from *MT‐ND5* to control region (Ishida et al. 2013). For both panels, S clade haplotypes (found only among savanna elephants) are within the rectangular box and F clade haplotypes (derived originally from forest elephants but present in some savanna elephants) are outside of the box. The number of nucleotide differences between connected haplotypes is one unless otherwise indicated by hatch marks or by a number. Haplotypes carried by Namibian desert elephants are shaded black, those carried by other Namibian elephants are in dark gray, while haplotypes carried by elephants from other countries (Fig. S3) are shown as light gray circles. Circle sizes are proportionate to the haplotype frequency in (B) and are numbered in descending order of frequency within Namibia (Table S3). For (A), frequencies were not available for all haplotypes but were available for all Namibian elephants (Table S4).

Novel control region sequences were combined with 316‐bp control region sequences generated by previous studies involving elephant mtDNA from across Africa (Eggert et al. 2002; Nyakaana et al. 2002; Debruyne et al. 2003; Debruyne 2005; Johnson et al. 2007; Ishida et al. 2011, 2013). A MJ network was constructed using elephant control region sequences from 81 locations across 22 countries in Africa (Fig. 2A), including 8 desert elephants, 8 elephants from the Caprivi Strip, and 72 elephants from other regions of Namibia. Namibian elephants carried both S and F clade haplotypes (Fig. 2). Previous analyses of African elephant mtDNA had identified a deep split into two clades: S clade found only in savanna elephants; and F clade found in all forest elephants (*Loxodonta cyclotis*) but also carried by many savanna elephants following mitochondrial interspecies transfer (Debruyne 2005; Roca et al. 2005). Within these two clades, eight distinctive subclades have been identified, three within the S clade and five within the F clade as shown in Figure 2, and most with limited regional distributions (Ishida et al. 2013).

In the Caprivi Strip (Fig. 1), among eight elephants, there were four haplotypes identified, previously designated H74 to H77 (Fig. 2A; Nyakaana et al. 2002; Johnson et al. 2007). Three of the haplotypes were part of the “Savanna‐wide” subclade of the S clade, which as its name suggests is a subclade distributed across African regions outside the tropical forest. The other haplotype was part of the “south‐central” subclade of the F clade, which extends also into Botswana and Zimbabwe (Roca et al. 2005; Ishida et al. 2013). These four haplotypes were not found elsewhere in Namibia, and indeed across Africa, they have been detected only in the Caprivi Strip.

Outside the Caprivi Strip, 80 elephants carried four distinct haplotypes (Figs. 1, 2, Table S4). These four haplotypes had been previously designated H03, H58, H62, and H63 (Johnson et al. 2007). Three of these fell within the “savanna‐wide” subclade: Haplotype 63 was detected only among elephants in Namibia, including four desert elephants and 28 elephants from other localities. Haplotype 62 is widely distributed in Africa (Ishida et al. 2013) and 5 desert and 41 other Namibian elephants carried this haplotype. Haplotype 58 was found in a single Namibian elephant in central Kunene and has been identified in Kenyan and Tanzanian elephants (Ishida et al. 2013). Haplotypes are not typically distributed so broadly (Ishida et al. 2013), and it is possible that the single mutation that defines this haplotype may represent a homoplasy. The fourth haplotype, H03, was part of the “south‐central” subclade of the mitochondrial F clade. It was carried by only two elephants in Namibia, neither of them desert dwellers.

The mitochondrial control region data from Namibia was grouped by geographic location as noted above (Caprivi Strip, desert elephants, central Kunene, Etosha NP, Huab, Ugab). The control region haplotype frequencies for each of these geographic regions are listed in Table S4 and displayed in Figure 1. For each pair of populations, *F*_ST_ and population differentiation were calculated (Table 1). The values of *F*_ST_ were high and significant when Caprivi Strip elephants were compared to those of any other Namibian locality; the values were low and not significant when any two populations within Namibia outside of Caprivi were compared. Population differentiation tests also determined that control region haplotypes of elephants in Caprivi were significantly different from those of any other Namibian location in pairwise comparisons (Table 1). The only exception was due to the low sample size for the central Kunene region, which led to a marginal *P*‐value for the population differentiation test between Caprivi Strip and central Kunene (*P *=* *0.0043), after Bonferroni correction set the level for significance to *α* < 0.0033.

**Table 1:** Population differentiation and *F*_ST_ between Namibian elephants

	Caprivi	Desert	C. Kunene	Etosha	Ugab	Huab
Caprivi	–	**	*NS*	**	**	*
Desert	0.41*	–	*NS*	*NS*	*NS*	*NS*
C. Kunene	0.46*	0.05	–	*NS*	*NS*	*NS*
Etosha	0.44**	0.00	0.24	–	*NS*	*NS*
Ugab	0.68**	0.29	0.10	0.36	–	*NS*
Huab	0.45*	0.00	0.00	0.15	0.07	–

Analyses are based on 316‐bp mtDNA control region sequences. Results of exact tests of population differentiation are above the diagonal, and *F*_ST_ results are below the diagonal.

Localities: Caprivi is the Caprivi Strip; Desert elephants are from the Hoanib or Hoarusib River catchments; C. Kunene refers to central Kunene; Etosha refers to Etosha National Park and nearby regions; Huab and Ugab refer to the catchments for the two rivers.

*indicates *P *<* *0.05, **indicates *P *<* *0.01, and *NS* indicates “not significant” (Bonferroni corrections applied).

Importantly, the eight desert elephants did not carry any unique control region haplotypes, and the desert elephants did not show significant differences from other Namibian populations (excluding Caprivi) in the *F*_ST_ calculation or in the population differentiation tests. When the other Namibian populations (except Caprivi) were combined into a single “nondesert” grouping (*n *=* *72) and compared to the group of 8 desert elephants, the population differentiation test was not significant, and *F*_ST_ was also not significant, with the value for *F*_ST_ estimated as zero. However, *F*_ST_ between Caprivi and the combined “nondesert” Namibian elephant group was very high at 0.44 as was *F*_ST_ between Caprivi and desert elephants, estimated at 0.41.

A dataset of sequences from across Africa is also available (for a smaller number of localities) for 4258 bp of continuous mtDNA sequence from part of the *MT‐ND5* gene through part of the control region (Ishida et al. 2013). The quality of DNA from dung is too low for amplification of such a long region, and only three desert elephants could be sequenced across this long fragment of DNA, along with 57 nondesert elephants primarily from Etosha NP, with some from nearby regions (Ishida et al. 2013). A MJ network analysis of the 4258‐bp alignment shows the relationships among the eight mtDNA subclades previously established across Africa (Fig. 2B; Ishida et al. 2013), and the placement of elephants from Etosha and of desert elephants in this network; these long sequences were not available for Caprivi, Huab, or Ugab elephants. Nine haplotypes were identified among the 60 Namibian elephants (Fig. 2B; Table S3). Eight of the nine haplotypes (designated 1–8 in Fig. 2B) were part of the Savanna‐wide subclade, while only one haplotype (numbered 9 in Fig. 2B) belonged to south‐central subclade within F clade. Among the three desert‐adapted elephants, one carried haplotype 4 (Fig. 2B, supplementary Table S3), which is common across African savanna localities and which was also carried by seven elephants in Etosha. The other two desert‐adapted elephants carried the Namibian‐specific haplotype 1, which had the highest frequency among Namibian elephants and was carried by 14 elephants in Etosha. The other haplotypes in Etosha (designated 2, 3, 5–9) were specific to Namibia and were not detected among elephants in other African countries. Thus, the longer mitochondrial sequences detected many haplotypes unique to northern Namibia. Although only limited information was provided by the three desert elephants that could be sequenced across the long mitochondrial DNA region, the two haplotypes that they carried were also common in Etosha elephants.

### Microsatellite analyses

As the mitochondrial analyses suggested that elephants across Namibia (outside Caprivi) had similar haplotypes in similar frequencies, we examined nuclear DNA markers in our Namibian elephant samples to determine whether nuclear gene flow was occurring across these populations. Twenty‐two microsatellite loci were genotyped in the higher quality Namibian samples, including 4 desert elephants and 51 Etosha elephants (50 from Etosha NP itself, with 1 from the Huab River catchment included as part of this Etosha set as a “nondesert” elephant; Table S2). In a conservative approach, five of the markers were removed due to monomorphism, deviation from Hardy–Weinberg expectations or LD. Genotypes of the remaining 17 microsatellite loci were used for subsequent analyses.

**Figure 3:**
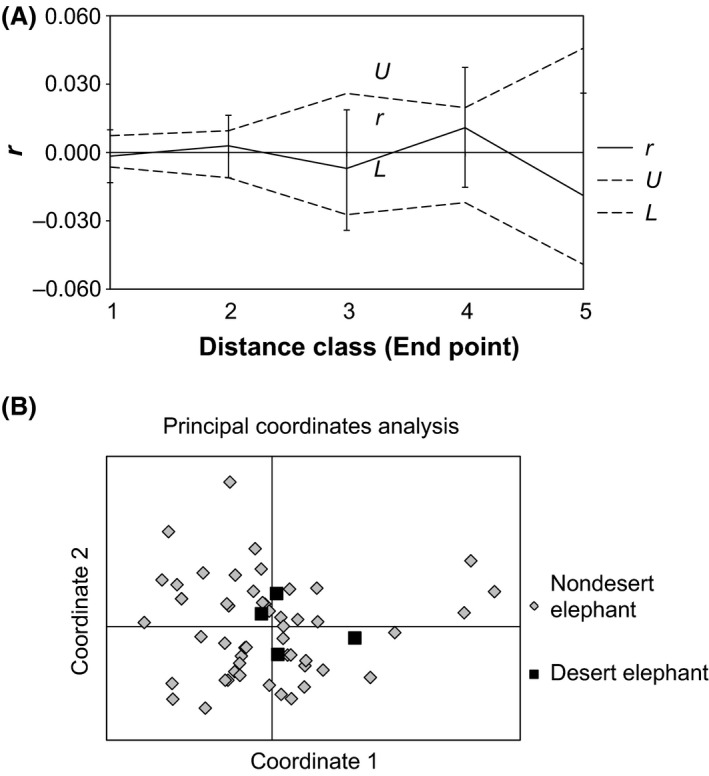
Genetic analyses of Namibian elephants using genotypes at 17 microsatellite loci. (A) Spatial genetic autocorrelograms of 55 Namibian elephants, implemented using the software GenAlEx 6.5 (Peakall and Smouse 2012). The genetic similarity between pairs of individuals (*y*‐axis) is shown relative to their geographic separation (*x*‐axis) (Peakall and Smouse 2012). The geographic distances between all possible pairs of individual were divided into quintiles (five groups each of the same size). *r*: spatial autocorrelation coefficient. *U*: upper 95% randomization limits of *r*. *L*: lower 95% randomization limits of *r*. In Namibian elephants, spatial distance and genetic distance were not correlated and isolation by distance was not observed at any distance class. (B) Principal coordinate analysis showing the genetic relationship of Namibian desert elephants (*n *=* *4) to Etosha elephants (*n *=* *51) performed on the genetic distance matrix. Only the first and second coordinates are shown here; neither differentiated between desert elephants and Etosha elephants, nor did the third coordinate (not shown).

We conducted a spatial autocorrelation analysis to examine the association between the genetic differences between pairs of individuals and their geographic separation. For the 51 “nondesert” elephants, geographic distances based on the geographic coordinates were computed between each pair of elephants, with the distances placed into even quintiles (*x*‐axis in Fig. 3A). Genetic distances between pairs of elephants were also determined (*y*‐axis in Fig. 3A). Both permutation and bootstrap tests did not detect significant spatial genetic autocorrelations in Namibian elephants at any distance class (Fig. 3A). This indicated that gene flow across Namibian elephants was recent and sufficient enough to prevent the development of patterns that would have been suggestive of isolation by distance.

African elephants exhibit female philopatry while males disperse from their natal social group (Archie et al. 2007; Hollister‐Smith et al. 2007). Thus, analyses were also conducted for males and for females separately to determine whether the spatial autocorrelation pattern would be different between the sexes. However, no autocorrelation was detected for either sex among Namibian elephants (Fig. S1). In this regard, it should be emphasized that although only males disperse, females receive half of their nuclear alleles from their fathers, so that any sex differences in nuclear genetic patterns would not persist in the presence of male‐mediated gene flow.

Although the number of high‐quality DNA samples for desert elephants was small, there were enough to compare their microsatellite genotypes to those of Etosha elephants. Between desert and Etosha elephants, *F*_ST_ was found to be low: *F*_ST_ was estimated as 0.035 using the software GenAlEx (Peakall and Smouse 2012), while using Arlequin (Excoffier and Lischer 2010) the value was estimated as zero (*P *=* *0.55). These estimates indicate that desert and “nondesert” elephants were quite similar in their nuclear genotypes. Likewise, an exact test of population differentiation conducted using Arlequin found no significant differentiation between Namibian desert and Etosha elephants (*P *>* *0.05). Neither did a PCoA distinguish desert from Etosha elephants for any of the first three coordinates (Fig. 3B; coordinate 3 not shown) although coordinates 1, 2, and 3 explained 24.15, 21.22, and 16.06% of the total variance, respectively. The program STRUCTURE did not place desert and Etosha elephants into different partitions (Fig. S2) whether all desert and Etosha elephants were examined together, or when the four desert elephants were compared to various sets each consisting of 4 randomly chosen Etosha elephants. Thus, all of the analyses of the microsatellite dataset found no differences between Namibian desert and Etosha elephants, suggesting that the two groups are genetically indistinguishable.

## Discussion

Most of the samples of desert elephants consisted of dung, from which DNA quality may be limited. We initially examined mitochondrial (mt) DNA patterns across Namibian elephants as successful amplification of DNA extracts from dung samples is more likely for mitochondrial than for nuclear markers. Additionally, mitochondrial DNA is transmitted only by female elephants, which typically are nondispersing. Finally, mtDNA has a high mutation rate, and given the matrilocality of elephant females, unique variants are found in many localities (Ishida et al. 2013). For these reasons, mtDNA haplotypes among elephants are very limited in geographic extent, and many localities have haplotype sequences that have not been detected elsewhere (Ishida et al. 2013). Thus, mtDNA would be expected to show differences across Namibian regions if the populations had been isolated for a substantial period of time. However, both types of DNA markers, mitochondrial DNA and nuclear microsatellite DNA, which provide information about different evolutionary trajectories (Nyakaana and Arctander 1999; Roca et al. 2005), showed that there is little or no genetic differentiation among desert elephants and other Namibian elephants outside of the Caprivi Strip.

There was mitochondrial DNA differentiation between the elephants of the Caprivi Strip and other Namibian elephants. This would seem consistent with previous reports that the elephants of southwest Angola and the Kaokoveld at the extreme northwest of Namibia together form a population that is somewhat isolated from elephants to their east in Caprivi and neighboring countries (Shortridge 1934; Martin 2005). The Caprivi elephants are likely different due to the relatively high geographic distances separating them from other Namibian localities (Fig. 1) or to migration from nearby regions in Angola, Botswana, Zambia, and Zimbabwe (Hoare 2004; Martin 2005). Note that we do not suggest that the Caprivi elephants are genetically distinctive (other than in mtDNA) from other elephants. Because elephant females do not disperse from their natal herds, mutations of mtDNA often are restricted to a single geographic locality (Ishida et al. 2013). Such locally restricted mitochondrial mutations would also account for the Caprivi elephants carrying haplotypes different from those previously reported (Ishida et al. 2013) from Chobe or Savuti in Botswana (see Fig. S3 for a map of all localities from which previous mitochondrial haplotypes are available). However, the dispersal of males prevents nuclear genetic distinctiveness from developing between localities that develop differences in mtDNA (Ishida et al. 2013). (Note: unless otherwise noted, subsequent statements regarding Namibian elephants exclude the elephant population in the Caprivi Strip.)

Previous analyses of nuclear genetic datasets have established that Etosha elephants are savanna elephants with low levels of nuclear genetic differentiation from other savanna populations (Roca et al. 2001, 2005; Ishida et al. 2011). The elephants of Etosha NP, the Huab River catchment, the Ugab River catchment, and our “Central Kunene” group were genetically indistinguishable from each other and from desert elephants. Given this lack of genetic differentiation, the desert elephants may be more accurately designated “desert‐dwelling elephants.” Previous studies have demonstrated that most elephant mtDNA haplotypes are locally or regionally limited in geographic range due to female matrilocality (Ishida et al. 2013). The absence of distinct mtDNA haplotypes in any of these Namibian regions and the similarity of mtDNA haplotype frequencies suggest that female migration across the localities has occurred relatively recently.

Although there was no genetic structuring of mtDNA or nuclear markers across elephant populations in Namibia, there was some evidence that Namibian elephants were somewhat genetically isolated from other savanna elephants. In a 4258‐bp mtDNA alignment, eight of nine haplotypes detected in Namibia were not detected in other African localities (Table S3). The Namibian‐specific haplotypes suggest that elephants have been present in Namibia for a long enough period to develop mtDNA distinctions. Rangewide patterns of genetic diversity in many species may reflect the effects of past climate change rather instead of following patterns expected based on whether populations are core or peripheral (Hampe and Petit 2005). It seems likely that elephant ranges would expand and contract quickly due to climate and habitat changes, as they are a highly vagile species (Parmesan 2006). Reconstructed lake‐level changes at Etosha Pan suggested that the climate was drier than it currently is prior to 8000 years ago, with various intervals since then when the climate was wetter than today (Brook et al. 2007). A period of marked aridity may have affected the region from 3500 until ca. 300 calendar years before present (Chase et al. 2009). It is unclear whether elephants survived in this part of Namibia across the changes in climate, or whether they migrated from other regions during less arid times. However, the stark difference in mtDNA haplotypes (Figs. 1, 2) suggests that migrants into Namibia did not originate from the population currently in the Caprivi Strip and its bordering countries.

Periods of drought, hunting, local extirpation, and subsequent immigration and expansion of elephants in different localities in Namibia may also have erased any genetic structure across populations. Some 300 years ago, savanna elephants ranged across most of Namibia at low densities wherever surface water was available during the dry season (Martin 2005). Due to commercial hunting and human settlement in the 1800s, elephant populations rapidly decreased in central and southern Namibia (Blanc et al. 2003; Martin 2005). By 1881, elephants had been eliminated in what is now Etosha NP (Berry 1997; Martin 2005), while the last Ugab elephant was shot in 1923 (Tommy Hall, personal communication to KL). A ground census found no elephants in Etosha Pan in 1926, although 40–50 elephants were counted in Ovamboland (Berry 1997; Martin 2005). By 1934, elephants were limited to Caprivi and the Kaokoveld (extreme northwest of Namibia; Kunene Region); there were just a few in Caprivi and a few vagrants in Outjo District, Ovamboland, and Okavango (Shortridge 1934; Martin 2005). At that time, Kaokoveld elephants were widely distributed from the Kunene River in the north to the Ugab River in the south and numbered from 600 to 1000 (Shortridge 1934; Martin 2005). Elephants began recolonizing Etosha in the 1950s, with 50–60 reported in 1952 (Berry 1997; Martin 2005). The Kunene population decreased during the independence war in Namibia, especially in the 1980s, a period during which these elephants were also affected by drought and increased poaching, reducing their numbers from 600 to 800 in 1968 to 250 by the late 1980s (Martin 2005). Elephant herds in the middle part of the Hoarusib River catchment, which had numbered 100 individuals in the 1950s, were wiped out (Viljoen 1987). In 1983, 357 elephants were counted in a survey of the Kaokoveld, of which 70 were desert‐dwelling elephants (Viljoen 1987, 1989a). The severe hunting of desert elephants appears to have disrupted normal social structure, as some social groups consist of unrelated females (Leggett et al. 2011). By contrast, the Etosha population rose from 500 in 1967 to a total of 2800 in 1983 (Lindeque 1988; Martin 2005). This change is too high to be explained by natural increase of the Etosha population and suggests high rates of migration from outside the park (Lindeque 1988; Martin 2005). Subsequent years saw large fluctuations in the Etosha population due to culling and to emigration or immigration in response to changes in rainfall and in hunting pressure outside the park (Martin 2005). In 2011, the number of elephants in Etosha was estimated by aerial sample survey as 3378 (http://www.elephantdatabase.org/population_submissions/198). With the end of the war in 1990, the number of elephants outside Etosha has also recovered, including the number of desert‐dwelling elephants (Viljoen 1992). Elephants moved from the Hoanib River catchment to the Hoarusib River catchment in 1997, with substantial movements occurring beginning in 2001 (Leggett et al. 2003). The range of elephants in Namibia has expanded southward to again include the Ugab River catchment (Martin 2005). Among our Ugab samples, there was only one mtDNA haplotype, which was also the most common haplotype in the Huab elephant population (Fig. 1), consistent with the movement of elephants from the Huab River catchment to the Ugab River catchment.

The history and mobility of Namibian elephants could account for the patterns detected using DNA. These factors would explain the genetic similarities between Etosha and other Namibian populations in their mtDNA (Fig. 1) and also help to explain the lack of isolation by distance (Fig. 3A) in Etosha, and lack of genetic structuring between desert‐dwelling elephants and Etosha elephants (Fig. 3B). There was no correlation between genetic and geographic distances among elephants in Namibia (Fig. 3A, B), which might be due to the recent expansion in the size and range of the elephant population (Martin 2005), to the large home range size of elephants in Namibia (2851–18,681 km^2^; Lindeque and Lindeque 1991), their long‐distance migration (Leggett 2006), and high levels of nuclear gene flow due to male dispersal. Although Etosha was said to be recolonized from both west and east (Martin 2005), current mtDNA patterns (Fig. 1) suggest that the Etosha elephants are not similar to those to the east in Caprivi (or to the Botswana population bordering Caprivi) but are similar to those to the west in the Kaokoveld.

The high gene flow and lack of evidence for isolation by distance in Namibia would suggest that phenotypic differences between desert and Etosha elephants would be unlikely to have evolved or persisted; there has not even been enough time or isolation for mtDNA haplotypes to differentiate between the two populations. The maximum body size for Etosha elephants is the same as those of other savanna elephants (Trimble et al. 2011). Likewise, the reported phenotypic differences among desert elephants are not well quantified and may be somewhat anecdotal. It may also be possible for a correlation between phenotype and environment to be due to the direct influence of environmental factors on the development of individual phenotypes (Chevin and Lande 2011). Such phenotypic plasticity would not depend on genetic differences between populations (Chevin and Lande 2011) and may account for the reported morphological characteristics of desert‐dwelling elephants. In summary, desert elephants do not represent the genetic adaptation of a species in a marginal environment. Instead, changes in behavior have allowed them to survive in a marginal environment and to survive a history of human encroachment, hunting, and warfare. Notably, migrations between Etosha and points west appear to have increased or decreased, or changed in direction, depending partly on climatic factors and also on hunting (including poaching) pressure placed on the elephants. Such learned behaviors can buffer a species against the effects of natural selection, lowering the pressure for local genetic adaptations to develop (Sutter and Kawecki 2009).

Although Namibian desert‐dwelling elephants are not genetically distinctive from other elephant populations in Namibia, there are other reasons to conserve them. The presence of elephants in the desert may be advantageous to the local environment as elephants open paths and dig for water underground, making these resources available to other animals (Viljoen 1992). Elephants facilitate the germination of seeds that they ingest; their deep tracks in the mud during the short rainy season are said to provide an ideal environment for seedlings (Viljoen 1992). Overhunting can have exceptionally severe impacts on large mammal population in extreme environments at low density (Petersen et al. 2010), especially given that desert‐dwelling elephants have a lower reproductive rate than other populations of savanna elephants (Leggett et al. 2011). If desert elephants were extirpated, they might not readily be replaced by other savanna elephants that had not learned the behaviors needed for desert survival. Thus, despite their genetic similarity to other savanna elephants, the desert‐dwelling elephants play an irreplaceable role in the desert ecosystem, which would justify conservation management to ensure their continued survival.

## Conflict of Interest

None declared.

## Data Accessibility

Mitochondrial DNA sequences: GenBank accession numbers are listed in Table S1, Supporting information.

## Supporting information


**Table S1.** Samples used in this study.
**Table S2.** Characterization of microsatellite loci genotyped in Namibian elephants.
**Table S3.** Haplotypes identified in Namibian elephants using 4258 bp mitochondrial DNA sequences.
**Table S4.** Haplotypes identified in Namibian elephants using 316 bp mitochondrial DNA sequences.
**Figure S1.** Spatial autocorrelation results for males (*n *=* *39) and females (*n *=* *11) were conducted separately using the software GenAlEx 6.5 (Peakall and Smouse 2012).
**Figure S2.** (A) Structure analysis (Pritchard et al. 2000) using 17 microsatellite genotypes of 55 Namibian elephants did not partition the dataset between desert and other elephants. (B) For the dataset of 17 microsatellites genotypes, STRUCTURE analyses were also conducted comparing the 4 desert elephants to 4 randomly chosen Etosha elephants, finding no distinction.
**Figure S3.** Map showing the geographic distribution of elephant sampling locations across Africa for which mtDNA sequences were available for comparison with the current dataset.
