# Supplementary material for: Genetic connectivity across marginal habitats: the elephants of the Namib Desert
Source: Ecol Evol. 2016 Aug 3;6(17):6189–201. doi: 10.1002/ece3.2352 (PMC5016642; doi:10.1002/ece3.2352)
Supplement: Supplementary file 1 — Table S1. Samples used in this study. Table S2. Characterization of microsatellite loci genotyped in Namibian elephants. Table S3. Haplotypes identified in Namibian elephants using 4258 bp mitochondrial DNA sequences. Table S4. Haplotypes identified in Namibian elephants using 316 bp mitochondrial DNA sequences. Figure S1. Spatial autocorrelation results for males (n = 39) and females (n = 11) were conducted separately using the software GenAlEx 6.5 (Peakall and Smouse 2012). Figure S2. (A) Structure analysis (Pritchard et al. 2000) using 17 microsatellite genotypes of 55 Namibian elephants did not partition the dataset between desert and other elephants. (B) For the dataset of 17 microsatellites genotypes, STRUCTURE analyses were also conducted comparing the 4 desert elephants to 4 randomly chosen Etosha elephants, finding no distinction. Figure S3. Map showing the geographic distribution of elephant sampling locations across Africa for which mtDNA sequences were available for comparison with the current dataset. [file ECE3-6-6189-s001.docx]

**Genetic connectivity across marginal habitats: the elephants of the Namib Desert**

Yasuko Ishida* (1), Peter J. Van Coeverden de Groot (2), Keith E. A. Leggett (3,4), Andrea S. Putnam (5), Virginia E. Fox (6), Jesse Lai (2), Peter T. Boag (2), Nicholas J. Georgiadis (7) and Alfred L. Roca* (1,8)

(1) Department of Animal Sciences, University of Illinois at Urbana-Champaign, Urbana IL 61801 USA

(2) Department of Biology, Queen's University, Kingston, ON K7L 3N6 Canada

(3) Namibian Elephant and Giraffe Trust, Outjo, Namibia

(4) Current address: Fowlers Gap Arid Zone Research Station; School of Biological, Earth and Environmental Sciences; University of New South Wales; Kensington, NSW 2051, Australia.

(5) Department of Life Sciences, San Diego Zoo Global, San Diego, CA 92112 USA

(6) Elephant Human Relations Aid (EHRA), Swakopmund, Namibia

(7) Puget Sound Institute, University of Washington, Tacoma, WA 98421 USA

(8) Carl R. Woese Institute for Genomic Biology, University of Illinois at Urbana-Champaign, Urbana IL 61801 USA

**Supporting information**

**Supporting information references**

Archie EA, Moss CJ, Alberts SC (2003) Characterization of tetranucleotide microsatellite loci in the African Savannah Elephant (*Loxodonta africana africana*). *Molecular Ecology Notes* **3**, 244-246.

Barriel V, Thuet E, Tassy P (1999) Molecular phylogeny of Elephantidae. Extreme divergence of the extant forest African elephant. *C R Acad Sci III* **322**, 447-454.

Comstock KE, Georgiadis N, Pecon-Slattery J*, et al.* (2002) Patterns of molecular genetic variation among African elephant populations. *Mol Ecol* **11**, 2489-2498.

Comstock KE, Wasser SK, Ostrander EA (2000) Polymorphic microsatellite DNA loci identified in the African elephant (*Loxodonta africana*). *Mol Ecol* **9**, 1004-1006.

Debruyne R (2005) A case study of apparent conflict between molecular phylogenies: the interrelationships of African elephants. *Cladistics* **21**, 31-50.

Debruyne R, Barriel V, Tassy P (2003) Mitochondrial cytochrome b of the Lyakhov mammoth (Proboscidea, Mammalia): new data and phylogenetic analyses of Elephantidae. *Mol Phylogenet Evol* **26**, 421-434.

Eggert LS, Rasner CA, Woodruff DS (2002) The evolution and phylogeography of the African elephant inferred from mitochondrial DNA sequence and nuclear microsatellite markers. *Proc R Soc Lond B Biol Sci* **269**, 1993-2006.

Fernando PJ, Vidya TNC, Melnick DJ (2001) Isolation and characterization of tri- and tetranucleotide microsatellite loci in the Asian elephant, *Elephas maximus*. *Molecular Ecology Notes* **1**, 232-233.

Ishida Y, Oleksyk TK, Georgiadis NJ*, et al.* (2011) Reconciling apparent conflicts between mitochondrial and nuclear phylogenies in African elephants. *PLoS One* **6**, e20642.

Ishida Y, Georgiadis NJ, Hondo T, Roca AL (2013) Triangulating the provenance of African elephants using mitochondrial DNA. *Evol Appl* **6**, 253-265.

Johnson MB, Clifford SL, Goossens B*, et al.* (2007) Complex phylogeographic history of central African forest elephants and its implications for taxonomy. *BMC Evol Biol* **7**, 244.

Nyakaana S, Arctander P (1998) Isolation and characterization of microsatellite loci in the African elephant, *Loxodonta africana*. *Mol Ecol* **7**, 1436-1437.

Nyakaana S, Arctander P, Siegismund HR (2002) Population structure of the African savannah elephant inferred from mitochondrial control region sequences and nuclear microsatellite loci. *Heredity* **89**, 90-98.

Peakall R, Smouse PE (2012) GenAlEx 6.5: genetic analysis in Excel. Population genetic software for teaching and research--an update. *Bioinformatics* **28**, 2537-2539.

Pritchard JK, Stephens M, Donnelly P (2000) Inference of population structure using multilocus genotype data. *Genetics* **155**, 945-959.

| **Supplementary Table S1. Samples used in this study.** | | | | | |  |  |  |  |  |
| --- | --- | --- | --- | --- | --- | --- | --- | --- | --- | --- |
|  |  |  |  |  |  |  |  |  |  |  |
| Sample ID | Sex | Desert or not | Collection date | Sample | Group | CR haplotype | GenBank No. | Coordinates | | Sample collection location |
| NA4651 | F | (not desert) | 1994 | Tissue | Etosha | H63 | JQ438544 | -19.2043 | 16.191933 | Aus Fountain, Etosha National Park |
| NA4652 | F | (not desert) | 1994 | Tissue | Etosha | H63 | JQ438545 | -19.2043 | 16.191933 | Aus Fountain, Etosha National Park |
| NA4653 | M | (not desert) | 1994 | Tissue | Etosha | H63 | JQ438546 | -19.2043 | 16.191933 | Aus Fountain, Etosha National Park |
| NA4655 | M | (not desert) | 1994 | Tissue | Etosha | H62 | JQ438547 | -19.2379 | 16.121017 | Oilfantsbad, Etosha |
| NA4656 | F | (not desert) | 1994 | Tissue | Etosha | H63 | JQ438548 | -19.2379 | 16.121017 | Oilfantsbad, Etosha |
| NA4657 | M | (not desert) | 1994 | Tissue | Etosha | H63 | JQ438549 | -19.2379 | 16.121017 | Oilfantsbad, Etosha |
| NA4658 | F | (not desert) | 1994 | Tissue | Etosha | H63 | JQ438550 | -19.2379 | 16.121017 | Oilfantsbad, Etosha |
| NA4659 | M | (not desert) | 1994 | Tissue | Etosha | H63 | JQ438551 | -19.2379 | 16.121017 | Oilfantsbad, Etosha |
| NA4660 | M | (not desert) | 1994 | Tissue | Etosha | H63 | JQ438552 | -19.2043 | 16.191933 | Aus Fountain, Etosha National Park |
| NA4661 | F | (not desert) | 1994 | Tissue | Etosha | H63 | JQ438553 | -19.2379 | 16.121017 | Oilfantsbad, Etosha |
| NA4662 | M | (not desert) | 1994 | Tissue | Etosha | H62 | JQ438554 | -19.2043 | 16.191933 | Aus Fountain, Etosha National Park |
| NA4663 | F | (not desert) | 1994 | Tissue | Etosha | H62 | JQ438555 | -19.2043 | 16.191933 | Aus Fountain, Etosha National Park |
| NA4664 | M | (not desert) | 1994 | Tissue | Etosha | H63 | JQ438556 | -19.2043 | 16.191933 | Aus Fountain, Etosha National Park |
| NA4665 | M | (not desert) | 1994 | Tissue | Etosha | H63 | JQ438557 | -19.2043 | 16.191933 | Aus Fountain, Etosha National Park |
| NA4666 | F | (not desert) | 1994 | Tissue | Etosha | H63 | JQ438558 | -19.2043 | 16.191933 | Aus Fountain, Etosha National Park |
| NA4667 | M | (not desert) | 1994 | Tissue | Etosha | H62 | JQ438559 | -18.586367 | 16.887167 | Mashara Lodge, 8 km from the Lindequist Gate of the Etosha National Park |
| NA4668 | M | (not desert) | 1994 | Tissue | Etosha | H63 | JQ438560 | -18.586367 | 16.887167 | Mashara Lodge, 8 km from the Lindequist Gate of the Etosha National Park |
| NA4669 | M | (not desert) | 1994 | Tissue | Etosha | H62 | JQ438561 | -18.586367 | 16.887167 | Mashara Lodge, 8 km from the Lindequist Gate of the Etosha National Park |
| NA4670 | M | (not desert) | 1994 | Tissue | Etosha | H62 | JQ438562 | -18.586367 | 16.887167 | Mashara Lodge, 8 km from the Lindequist Gate of the Etosha National Park |
| NA4671 | M | (not desert) | 1994 | Tissue | Etosha | H62 | JQ438563 | -18.586367 | 16.887167 | Mashara Lodge, 8 km from the Lindequist Gate of the Etosha National Park |
| NA4672 | F | (not desert) | 1994 | Tissue | Etosha | H62 | JQ438564 | -18.586367 | 16.887167 | Mashara Lodge, 8 km from the Lindequist Gate of the Etosha National Park |
| NA4673 | M | (not desert) | 1994 | Tissue | Etosha | H63 | JQ438565 | -18.586367 | 16.887167 | Mashara Lodge, 8 km from the Lindequist Gate of the Etosha National Park |
| NA4674 | F | (not desert) | 1994 | Tissue | Etosha | H62 | JQ438566 | -18.6175 | 16.988883 | Kameeldoring Pan, Etosha National Park |
| NA4675 | M | (not desert) | 1994 | Tissue | Etosha | H63 | JQ438567 | -18.586367 | 16.887167 | Mashara Lodge, 8 km from the Lindequist Gate of the Etosha National Park |
| NA4677 | M | (not desert) | 1994 | Tissue | Etosha | H62 | JQ438568 | -18.6175 | 16.988883 | Kameeldoring Pan, Etosha National Park |
| NA4678 | M | (not desert) | 1994 | Tissue | Etosha | H62 | JQ438569 | -18.6175 | 16.988883 | Kameeldoring Pan, Etosha National Park |
| NA4679 | N/A | (not desert) | 1994 | Tissue | Etosha | H63 | JQ438570 | -18.6175 | 16.988883 | Kameeldoring Pan, Etosha National Park |
| NA4680 | M | (not desert) | 1994 | Tissue | Etosha | H63 | JQ438571 | -18.942617 | 16.7017 | Batia, Etosha National Park |
| NA4681 | M | (not desert) | 1994 | Tissue | Etosha | H62 | JQ438572 | -18.942617 | 16.7017 | Batia, Etosha National Park |
| NA4685 | F | (not desert) | 1994 | Tissue | Huab River | H62 | JQ438573 | -20.363681 | 14.937762 | Khorixas |
| NA4686 | F | (not desert) | 1994 | Tissue | Huab River | H62 | JQ438574 | -20.372065 | 14.959329 | Damara Mopane Lodge |
| NA4687 | F | (not desert) | 1994 | Tissue | C. Kunene | H62 | JQ438575 | -19.857517 | 13.843031 | Krone Canyon |
| NA4688 | M | (not desert) | 1994 | Tissue | C. Kunene | H62 | JQ438576 | -19.857517 | 13.843031 | Krone Canyon |
| NA4689 | M | (not desert) | 1994 | Tissue | Etosha | H62 | JQ438577 | -19.174183 | 15.914417 | Okakukejo, Kunene |
| NA4690 | M | (not desert) | 1994 | Tissue | Etosha | H63 | JQ438578 | -19.174183 | 15.914417 | Okakukejo, Kunene |
| NA4691 | M | (not desert) | 1994 | Tissue | Etosha | H62 | JQ438579 | -19.174183 | 15.914417 | Okakukejo, Kunene |
| NA4692 | M | (not desert) | 1994 | Tissue | Etosha | H63 | JQ438580 | -19.174183 | 15.914417 | Okakukejo, Kunene |
| NA4695 | M | (not desert) | 1994 | Tissue | Etosha | H62 | JQ438581 | -19.174183 | 15.914417 | Okakukejo, Kunene |
| NA4696 | M | (not desert) | 1994 | Tissue | Etosha | H63 | JQ438582 | -19.174183 | 15.914417 | Okakukejo, Kunene |
| NA4697 | M | (not desert) | 1994 | Tissue | Etosha | H62 | JQ438583 | -19.174183 | 15.914417 | Okakukejo, Kunene |
| NA4698 | M | (not desert) | 1994 | Tissue | Etosha | H63 | JQ438584 | -19.174183 | 15.914417 | Okakukejo, Kunene |
| NA4699 | M | (not desert) | 1994 | Tissue | C. Kunene | H62 | JQ438585 | -19.627183 | 14.842543 | Kamanjab |
| NA4701 | N/A | (not desert) | 1994 | Tissue | Etosha |  |  | -19.033333 | 16.483333 | Helio, National Park |
| NA4702 | M | (not desert) | 1994 | Tissue | Etosha | H63 | JQ438586 | -19.2379 | 16.121017 | Oilfantsbad, Etosha |
| NA4703 | F | (not desert) | 1994 | Tissue | Etosha | H62 | JQ438587 | -18.674494 | 15.577669 | North of Pan Point, Etosha National Park |
| NA4704 | M | (not desert) | 1994 | Tissue | Etosha | H03 | JQ438588 | -18.78333 | 15.583333 | Pan Point, Etosha Nationa Park |
| NA4705 | M | (not desert) | 1994 | Tissue | Etosha | H62 | JQ438589 | -18.78333 | 15.583333 | Pan Point, Etosha Nationa Park |
| NA4706 | M | (not desert) | 1994 | Tissue | Etosha | H63 | JQ438590 | -18.966667 | 15.31667 | Sonderkop, Etosha Restricted Area |
| NA4707 | N/A | (not desert) | 1994 | Tissue | Etosha | H62 | JQ438591 | -18.966667 | 15.31667 | Sonderkop, Etosha Restricted Area |
| NA4708 | M | (not desert) | 1994 | Tissue | Etosha | H62 | JQ438592 | -18.966667 | 15.05 | Teespoed, Etosha Restricted Area |
| NA4709 | N/A | (not desert) | 1994 | Tissue | Etosha |  |  | -18.966667 | 15.05 | Teespoed, Etosha Restricted Area |
| NA4710 | M | (not desert) | 1994 | Tissue | Etosha | H62 | JQ438593 | -18.966667 | 15.05 | Teespoed, Etosha Restricted Area |
| NA4711 | N/A | (not desert) | 1994 | Tissue | Etosha |  |  | -18.956623 | 14.963722 | Tobiroen, Etosha National Park |
| NA4712 | N/A | (not desert) | 1994 | Tissue | Etosha |  |  | -18.956623 | 14.963722 | Tobiroen, Etosha National Park |
| NA4713 | M | (not desert) | 1994 | Tissue | Etosha |  |  | -18.85 | 15.03333 | Nerens, Etosha National Park |
| NA4716 | N/A | (not desert) | 1994 | Tissue | C. Kunene | H58 | JQ438594 | -19.316667 | 13.966667 | Khowarib Schlucht, Kunene, Namibia |
| NA4717 | N/A | (not desert) | 1994 | Tissue | C. Kunene | H62 | JQ438595 | -19.316667 | 13.966667 | Khowarib Schlucht, Kunene, Namibia |
| NA4720 | N/A | (not desert) | 1994 | Tissue | Etosha | H62 | JQ438596 |  |  | Etosha |
| NA4721 | F | (not desert) | 1994 | Tissue | Etosha | H63 | JQ438597 | -19.213434 | 16.059196 | Gemsbokvlakte, Oshikoto |
| Sample ID | Sex | Desert or not | Collection date | Sample | Group | CR haplotype | GenBank No. | Coordinates | | Sample collection location |
| NA4722 | M | (not desert) | 1994 | Tissue | Etosha | H63 | JQ438598 | -19.213434 | 16.059196 | Gemsbokvlakte, Oshikoto |
| NA5122 | N/A | Desert | 2002 | Dung | Desert | H62 | JQ438599 | -19.383333 | 13.1 | Hoanib River |
| NA5113 | N/A | Desert | 2002 | Dung | Desert | H63 | JQ438600 | -19.383333 | 13.1 | Hoanib River |
| NA5117 | N/A | Desert | 2002 | Dung | Desert | H62 | JQ438601 | -19.383333 | 13.1 | Hoanib River |
| NA5116 | N/A | Desert | 2002 | Dung | Desert | H63 | JQ438602 | -19.383333 | 13.1 | Hoanib River |
| NA5014 | M | (not desert) | 7/8/04 | Dung | Ugab River | H62 | JQ438603 | -20.941311 | 14.79774 | Ugab River |
| NA5015 | F | (not desert) | 7/8/04 | Dung | Ugab River | H62 | AF106235 | -20.941311 | 14.79774 | Ugab River |
| NA5016 | M | (not desert) | 7/8/04 | Dung | Ugab River | H62 | AF106236 | -20.941311 | 14.79774 | Ugab River |
| NA5017 | M | (not desert) | 6/6/04 | Dung | Ugab River | H62 | AF106237 | -20.941311 | 14.79774 | Ugab River |
| NA5018 | M | (not desert) | 6/6/04 | Dung | Ugab River | H62 | AF106238 | -20.941311 | 14.79774 | Ugab River |
| NA5019 | M | (not desert) | 9/6/04 | Dung | Ugab River | H62 | AF106211, AF527682, AY742800, AY741325, AY741323* | -20.941311 | 14.79774 | Ugab River |
| NA5020 | M | (not desert) | 9/6/04 | Dung | Ugab River | H62 | AF106211, AF527682, AY742800, AY741325, AY741323* | -20.941311 | 14.79774 | Ugab River |
| NA5022 | M | (not desert) | 9/8/04 | Dung | Huab River | H62 | AF106211, AF527682, AY742800, AY741325, AY741323* | -20.399175 | 15.00424 | Huab |
| NA5024 | M | (not desert) | 5/17/04 | Dung | Ugab River | H62 | AF106211, AF527682, AY742800, AY741325, AY741323* | -20.941311 | 14.79774 | Sorris-Sorris |
| NA5028 | M | (not desert) | 9/12/04 | Dung | Huab River | H62 | AF106211, AF527682, AY742800, AY741325, AY741323* | -20.439938 | 14.619192 | Aba-Huab River |
| NA5029 | M | (not desert) | 13/09/04 | Dung | Huab River | H63 | AF106239* | -20.439938 | 14.619192 | Aba-Huab River |
| NA5030 | F | (not desert) | 9/13/04 | Dung | Huab River | H62 | AF106211, AF527682, AY742800, AY741325, AY741323* | -20.439938 | 14.619192 | Aba-Huab River |
| NA5032 | F | (not desert) | 9/14/04 | Dung | Huab River | H62 | AF106211, AF527682, AY742800, AY741325, AY741323* | -20.399175 | 15.00424 | Huab |
| NA5099 | M | (not desert) | 3/14/03 | Dung | Huab River | H03 | AF106228, AF106234, AY359275* | -20.399175 | 15.00424 | Huab |
| NA5202 | M | (not desert) | 2002 | Blood | Etosha | H63 | AF106239* | -19.346838 | 14.40247 | Hobatere Concession, Kunene |
| NA5203 | M | (not desert) | 2002 | Blood | Etosha | H62 | AF106211, AF527682, AY742800, AY741325, AY741323* | -19.346838 | 14.40247 | Hobatere Concession, Kunene |
| NA5204 | F | (not desert) | 2002 | Blood | Etosha | H63 | AF106239* | -19.346838 | 14.40247 | Hobatere Concession, Kunene |
| NA5205 | M | Desert | 2002 | Blood | Desert | H62 | AF106211, AF527682, AY742800, AY741325, AY741323* | -18.773699 | 12.95075 | Purros |
| NA5206 | M | Desert | 2002 | Blood | Desert | H62 | AF106211, AF527682, AY742800, AY741325, AY741323* | -18.773699 | 12.95075 | Purros |
| NA5207 | M | Desert | 2002 | Blood | Desert | H63 | AF106239* | -18.773699 | 12.95075 | Purros |
| NA5208 | F | Desert | 2002 | Blood | Desert | H62 | AF106211, AF527682, AY742800, AY741325, AY741323* | -18.773699 | 12.95075 | Purros |
|  |  |  |  |  |  |  |  |  |  |  |
| CR (control region) haplotype designations follow those of Johnson et al. (2007). | | | | | | |  |  |  |  |
| *316 bp CR sequences matched with the previously reported sequences (Debruyne 2005; Debruyne et al. 2003; Eggert et al. 2002; Nyakaana et al. 2002) (listed in Johnson et al. 2007). | | | | | | | | | | |
|  |  |  |  |  |  |  |  |  |  |  |
|  |  |  |  |  |  |  |  |  |  |  |
|  |  |  |  |  |  |  |  |  |  |  |
|  |  |  |  |  |  |  |  |  |  |  |

| **Supplementary Table S2. Characterization of microsatellite loci genotyped in Namibian elephants.** | | | | | | | | | | | | | | | | | | | | | | |  |  |  |  |  |  |
| --- | --- | --- | --- | --- | --- | --- | --- | --- | --- | --- | --- | --- | --- | --- | --- | --- | --- | --- | --- | --- | --- | --- | --- | --- | --- | --- | --- | --- |
|  |  | | |  |  | |  |  |  | |  |  | |  | |  |  | |  |  |  |  |  |  |  |  |  |  |
|  | HWE | | |  | Total | | | | | |  | Non-desert localities | | | | | | |  | Desert elephants | | | |  |  |  |  |  |
| Locus | *p*-value | | |  | *n* | | Allele No. | *Ho* | *He* | |  | *n* | | Allele No. | | *Ho* | *He* | |  | *n* | Allele No. | *Ho* | *He* |  | References |  |  |  |
| LafMS03 | 0.188 | | |  | 55 | | 3 | 0.49 | 0.53 | |  | 51 | | 3 | | 0.47 | 0.53 | |  | 4 | 2 | 0.75 | 0.54 |  | Nyakaana and Arctander 1998 |  |  |  |
| LafMS04 | 0.623 | | |  | 55 | | 4 | 0.27 | 0.30 | |  | 51 | | 4 | | 0.25 | 0.29 | |  | 4 | 3 | 0.50 | 0.46 |  | Nyakaana and Arctander 1998 |  |  |  |
| FH39 | 0.720 | | |  | 55 | | 9 | 0.93 | 0.78 | |  | 51 | | 9 | | 0.92 | 0.78 | |  | 4 | 6 | 1.00 | 0.89 |  | Comstock et al. 2000 |  |  |  |
| FH40 | 0.106 | | |  | 55 | | 5 | 0.73 | 0.66 | |  | 51 | | 5 | | 0.73 | 0.67 | |  | 4 | 3 | 0.75 | 0.61 |  | Comstock et al. 2000 |  |  |  |
| FH48 | 0.345 | | |  | 55 | | 7 | 0.58 | 0.54 | |  | 51 | | 7 | | 0.57 | 0.52 | |  | 4 | 2 | 0.75 | 0.54 |  | Comstock et al. 2000 |  |  |  |
| FH60 | 1.000 | | |  | 55 | | 3 | 0.58 | 0.56 | |  | 51 | | 3 | | 0.57 | 0.57 | |  | 4 | 2 | 0.75 | 0.54 |  | Comstock et al. 2000 |  |  |  |
| FH67 | 0.530 | | |  | 55 | | 3 | 0.45 | 0.48 | |  | 51 | | 3 | | 0.43 | 0.48 | |  | 4 | 2 | 0.75 | 0.54 |  | Comstock et al. 2000 |  |  |  |
| FH71 | 0.013 | | |  | 55 | | 3 | 0.38 | 0.44 | |  | 51 | | 3 | | 0.37 | 0.45 | |  | 4 | 2 | 0.50 | 0.43 |  | Comstock et al. 2000 |  |  |  |
| FH94 | 0.575 | | |  | 54 | | 5 | 0.70 | 0.69 | |  | 50 | | 5 | | 0.72 | 0.70 | |  | 4 | 2 | 0.50 | 0.43 |  | Comstock et al. 2000 |  |  |  |
| FH102 | 0.704 | | |  | 52 | | 5 | 0.63 | 0.67 | |  | 48 | | 4 | | 0.63 | 0.67 | |  | 4 | 4 | 0.75 | 0.75 |  | Comstock et al. 2000 |  |  |  |
| EMX-4 | 1.000 | | |  | 54 | | 2 | 0.43 | 0.44 | |  | 51 | | 2 | | 0.41 | 0.44 | |  | 3 | 2 | 0.67 | 0.53 |  | Fernando et al. 2001 |  |  |  |
| FH127 | 0.774 | | |  | 54 | | 11 | 0.80 | 0.81 | |  | 50 | | 10 | | 0.78 | 0.82 | |  | 4 | 5 | 1.00 | 0.86 |  | Comstock et al. 2002 |  |  |  |
| FH129 | 0.428 | | |  | 53 | | 6 | 0.74 | 0.70 | |  | 49 | | 6 | | 0.73 | 0.71 | |  | 4 | 3 | 0.75 | 0.61 |  | Comstock et al. 2002 |  |  |  |
| FH153 | 0.654 | | |  | 54 | | 11 | 0.81 | 0.82 | |  | 50 | | 11 | | 0.82 | 0.82 | |  | 4 | 5 | 0.75 | 0.86 |  | Comstock et al. 2002 |  |  |  |
| LaT05 | 0.257 | | |  | 54 | | 13 | 0.85 | 0.88 | |  | 50 | | 12 | | 0.84 | 0.87 | |  | 4 | 7 | 1.00 | 0.96 |  | Archie et al. 2003 |  |  |  |
| LaT06 | 0.000* | | |  | – | | – | – | – | | – | – | | – | | – | – | | – | – | – | – | – |  | Archie et al. 2003 |  |  |  |
| LAF11 | 1.000 | | |  | 55 | | 2 | 0.35 | 0.33 | |  | 51 | | 2 | | 0.37 | 0.35 | |  | 4 | 2 | 0.25 | 0.25 |  | Ishida et al. 2011 |  |  |  |
| LAF13 | 1.000 | | |  | 55 | | 2 | 0.51 | 0.50 | |  | 51 | | 2 | | 0.49 | 0.50 | |  | 4 | 2 | 0.75 | 0.54 |  | Ishida et al. 2011 |  |  |  |
| LAF29 | 0.177 | | |  | 53 | | 6 | 0.64 | 0.63 | |  | 49 | | 6 | | 0.61 | 0.62 | |  | 4 | 4 | 1.00 | 0.75 |  | Ishida et al. 2011 |  |  |  |
| LAF37 | 0.094 | | |  | 54 | | 4 | 0.57 | 0.67 | |  | 50 | | 4 | | 0.56 | 0.67 | |  | 4 | 3 | 0.75 | 0.61 |  | Ishida et al. 2011 |  |  |  |
| FH19 | 0.000* | | |  | – | | – | – | – | | – | – | | – | | – | – | | – | – | – | – | – |  | Comstock et al. 2000 |  |  |  |
| EMX-5 | Monomorphic | | |  | – | | – | – | – | | – | – | | – | | – | – | | – | – | – | – | – |  | Fernando et al. 2001 |  |  |  |
|  |  | | |  |  | |  |  |  | |  |  | |  | |  |  | |  |  |  |  |  |  |  |  |  |  |
| Mean |  | | |  |  | | 5.47 | 0.60 | 0.60 | |  |  | | 5.32 | | 0.59 | 0.60 | |  |  | 3.21 | 0.73 | 0.61 |  |  |  |  |  |
| *s.d.* |  | | |  |  | | 3.32 | 0.18 | 0.16 | |  |  | | 3.13 | | 0.18 | 0.16 | |  |  | 1.55 | 0.20 | 0.19 |  |  |  |  |  |
|  |  | | |  |  | |  |  |  | |  |  | |  | |  |  | |  |  |  |  |  |  |  |  |  |  |
| Asterisks indicate markers showing significant deviation from HWE after Bonferroni correction (p < 0.002). Markers LaT06, FH19, and EMX-5 were removed from the subsequent population analyses. | | | | | | | | | | | | | | | | | | | | | | | | | |  |  |  |
|  |  |  |  |  |  |  |  |  |  |  |  |  |  |  |  |  |  |  |  |  |  |  |  |  |  |  |  |  |
| n: sample size, Ho: observed heterozygosity, He: expected heterozygosity. | | | | | | | | | | | | | | | | |  | |  |  |  |  |  |  |  |  |  |  |
|  | |  |  | | |  | | | |  | | |  | |  | | |  | | | | | | | |  |  |  |
|  | |  |  | | |  | | | |  | | |  | |  | | |  | | | | | | | |  |  |  |
|  | |  |  | | |  | | | |  | | |  | |  | | |  | | | | | | | |  |  |  |

| **Supplementary Table S3. Haplotypes identified in Namibian elephants using 4258 bp mtDNA sequences.** | | | | | |  |  |
| --- | --- | --- | --- | --- | --- | --- | --- |
| Haplotype |  | Namibia | |  | Other countries |  |  |
|  |  | Etosha | Desert |  |  |  |  |
| NA1 |  | 14 | 2 |  | 0 |  |  |
| NA2 |  | 13 | 0 |  | 0 |  |  |
| NA3 |  | 9 | 0 |  | 0 |  |  |
| NA4 |  | 7 | 1 |  | 41* |  |  |
| NA5 |  | 6 | 0 |  | 0 |  |  |
| NA6 |  | 5 | 0 |  | 0 |  |  |
| NA7 |  | 1 | 0 |  | 0 |  |  |
| NA8 |  | 1 | 0 |  | 0 |  |  |
| NA9 |  | 1 | 0 |  | 0 |  |  |
|  |  |  |  |  |  |  |  |
| Haplotype names follow those of Figure 2B. | | | | | | |  |
| *The NA4 haplotype was also detected in elephants from Aberdares (n = 1) in Kenya; Ngorongoro (*n* = 3) and Tarangire (*n* = 21); in Tanzania; Chobe (*n* = 12), Mashatu (*n* = 1), Savuti (*n* = 1) in Botswana; and Zambezi (*n* = 2) in Zimbabwe. | | | | | |  |  |
|  |  |  |  |  |  |  |  |
|  |  |  |  |  |  |  |  |
|  |  |  |  |  |  |  |  |
|  |  |  |  |  |  |  |  |
|  |  |  |  |  |  |  |  |

| **Supplementary Table S4. Haplotypes identified in Namibian elephants using 316 bp mitochondrial DNA sequences.** | | | | | | | | |  |  |  |  |
| --- | --- | --- | --- | --- | --- | --- | --- | --- | --- | --- | --- | --- |
| Haplotype |  | Caprivi | Desert | C. Kunene | Etosha | Huab | Ugab | Other countries* |  |  |  |  |
| H03 |  | 0 | 0 | 0 | 1 | 1 | 0 | Botswana, Zimbabwe |  |  |  |  |
| H58 |  | 0 | 0 | 1 | 0 | 0 | 0 | Kenya, Tanzania |  |  |  |  |
| H62 |  | 0 | 5 | 4 | 23 | 6 | 8 | Botswana, Kenya, South Africa,Tanzania, Uganda, Zimbabwe |  |  |  |  |
| H63 |  | 0 | 3 | 0 | 27 | 1 | 0 | none |  |  |  |  |
| H74 |  | 1 | 0 | 0 | 0 | 0 | 0 | none |  |  |  |  |
| H75 |  | 5 | 0 | 0 | 0 | 0 | 0 | none |  |  |  |  |
| H76 |  | 1 | 0 | 0 | 0 | 0 | 0 | none |  |  |  |  |
| H77 |  | 1 | 0 | 0 | 0 | 0 | 0 | none |  |  |  |  |
|  |  |  |  |  |  |  |  |  |  |  |  |  |
| Haplotype designations follow those of Johnson et al. (2007). | | | | | | | |  |  |  |  |  |
| The Caprivi Strip data is from Nyakaana et al. (2002). | | | | | | |  |  |  |  |  |  |
| *Countries other than Namibia in which the haplotype has been detected. | | | | | | | | |  |  |  |  |
|  |  |  |  |  |  |  |  |  |  |  |  |  |
|  |  |  |  |  |  |  |  |  |  |  |  |  |
|  |  |  |  |  |  |  |  |  |  |  |  |  |

**Figure S1.** Spatial autocorrelation results for males (*n* = 39) and females (*n* = 11) were conducted separately using the software GenAlEx 6.5 (Peakall & Smouse 2012). Samples for which the sex was unknown were not included in the analyses. Geographic distances were calculated for all pairs of elephants, then the geographic distances were divided into quintiles and the program was run using 999 random permutations and 1,000 bootstraps. *r*: spatial autocorrelation coefficient. *U*: upper 95% randomization limits of *r*. *L*: lower 95% randomization limits of *r*. Neither males nor females showed significant spatial autocorrelation.

**Figure S2.** (A) Structure analysis (Pritchard *et al.* 2000) using 17 microsatellite genotypes of 55 Namibian Etosha elephants did not partition the dataset between desert and other elephants. The program was run using values of *K* from 1 to 10. Each analysis was run for at least 1 million Markov chain Monte Carlo generations following a burn-in of at least 100,000 steps using an admixture-correlated model. The results are shown for *K* = 2, K = 3, and K = 4. (B) For the dataset of 17 microsatellites genotypes, STRUCTURE analyses were also conducted comparing the 4 desert elephants to 4 randomly chosen Etosha elephants. Results are shown for *K = 2* for several different sets of randomly chosen Etosha elephants.

**Figure S3. Map showing the geographic distribution of elephant sampling locations across Africa for which mtDNA sequences were available for comparison with the current dataset.** The map and associated information were previously published by Ishida et al. (2013) and are reproduced here as permitted under the terms of the Creative Commons Attribution License (http://onlinelibrary.wiley.com/journal/10.1111/(ISSN)1752-4571/homepage/open_access_license_and_copyright.htm).

Colors on the map represent mitochondrial DNA subclades as indicated in the key and described in Ishida et al. (2013). The large pie charts represent localities from which elephants have been sequenced for 4258 bp of mitochondrial DNA (from part of *ND5* to the control region), see Ishida et al. (2013). Locations in tropical forest habitats are: DS-Dzanga Sangha, Central African Republic; OD-Odzala, Republic of Congo; BF-Bili Forest, Democratic Republic of Congo; LO-Lope, Gabon; and SL-Sierra Leone (one zoo individual). Savanna locations: CH-Chobe, MA-Mashatu, SA-Savuti in Botswana; BE-Benoue, WA-Waza in Cameroon; AB-Aberdares, AM-Amboseli, KE-Central Kenya/Laikipia, MK-Mount Kenya in Kenya; NA-Northern Namibia/Etosha; KR-Kruger in South Africa; NG-Ngorongoro, SE-Serengeti, TA-Tarangire in Tanzania; HW-Hwange, SW-Sengwa, ZZ-Zambezi in Zimbabwe. GR-Garamba is located in the Guinea-Congolian/Sudanian transition zone of vegetation in D.R. Congo.

The small circles represent localities sampled by other previous mtDNA studies, from which mitochondrial control region sequences are available (Barriel et al. 1999; Eggert et al. 2002; Nyakaana et al. 2002; Debruyne et al. 2003; Debruyne 2005; Johnson et al. 2007); these are numbered as follows:
